# Supplementary material for: Prospective association between depressive symptoms and blood-pressure related outcomes in Kosovo
Source: PLOS Glob Public Health. 2023 Apr 7;3(4):e0000851. doi: 10.1371/journal.pgph.0000851 (PMC10081745; doi:10.1371/journal.pgph.0000851)
Supplement: S1 Table — mmHg: Millimetres of mercury, DASS: Depression Anxiety Stress Scale, BMI: Body mass index, SD: Standard deviation, IQR: Interquartile range. Normal to mild depressive symptoms if depression subscale of 21-item Depression Anxiety Stress Scale score was <14. Moderate to very severe depressive symptoms if depression subscale of 21-item Depression Anxiety Stress Scale score was ≥14. (DOCX) [file pgph.0000851.s001.docx]

S1 Table. Baseline characteristics disaggregated by participant and non-participant status

| **Sociodemographic factors** | **Participant**  **n=648** | **Non-participant**  **n =363** | |  |
| --- | --- | --- | --- | --- |
| Age, mean (SD) | 59.4 (8.9) | 60.4 (10.0) | p=0.087 ^a^ | |
| Sex, frequency (%)  Male  Female | 273 (42.1)  375 (57.9) | 140 (38.6)  223 (61.4) | p=0.269 ^b^ | |
| Education, frequency (%)  Primary school or less  Secondary school  University/College | 399 (61.6)  204 (31.5)  45 (6.9) | 239 (65.8)  108 (29.8)  16 (4.4) | p=0.185 ^b^ | |
| Work status, frequency (%)  Currently working  House person  Retired or disabled  Unemployed | 119 (18.4)  303 (46.8)  208 (32.0)  18 (2.8) | 52 (14.3)  175 (48.2)  116 (32.0)  20 (5.5) | p=0.073 ^b^ | |
| Residence, frequency (%)  Rural  Urban | 372 (57.4)  276 (42.6) | 202 (55.7)  161 (44.3) | p=0.588 ^b^ | |
| Municipality, frequency (%)  Drenas  Fushe Kosova  Gjakova  Gračanica  Junik  Lipjan  Malisheva  Mitrovica  Obiliq  Rahovec  Skenderaj  Vushtrri | 63 (9.7)  64 (9.9)  51 (7.9)  36 (5.6)  11 (1.7)  106 (16.4)  51 (7.9)  65 (10.0)  37 (5.7)  53 (8.2)  69 (10.7)  42 (6.5) | 40 (11.0)  46 (12.7)  23 (6.3)  19 (5.2)  11 (3.0)  71 (19.6)  29 (8.0)  19 (5.2)  33 (9.1)  24 (6.6)  29 (8.0)  19 (5.2) | p=0.054 ^b^ | |
| Ethnicity, frequency (%)  Albanian  Serbian  Roma, Ashkali, Egyptian, Other | 589 (90.9)  34 (5.2)  25 (3.9) | 331 (91.2)  17 (4.7)  15 (4.1) | p=0.908 ^b^ | |
| Main Family Medicine Center visits in the last 6 months, median (IQR) | 3 (2-6) | 3 (2-7) | p=0.685 ^a^ | |
| Smoking, frequency (%)  Never or ex-smoker  Current smoker | 521 (80.4)  127 (19.6) | 284 (78.2)  79 (21.8) | p=0.412 ^b^ | |
| Physical activity, frequency (%)  Sufficiently active  Insufficiently active | 211 (32.6)  437 (67.4) | 85 (23.4)  278 (76.6) | p=0.002 ^b^ | |
| Alcohol, frequency (%)  No alcohol in past 30 days  Consumed alcohol in past 30 days | 616 (95.1)  32 (4.9) | 350 (96.4)  13 (3.6) | p=0.316 ^b^ | |
| Nutrition, frequency (%)  Adequate nutrition  Poor nutrition | 98 (15.1)  550 (84.9) | 52 (14.3)  311 (85.7) | p=0.732 ^b^ | |
| Sleep, frequency (%)  Very good  Fairly good  Fairly bad  Very bad | 175 (27.0)  236 (36.4)  173 (26.7)  64 (9.9) | 102 (28.1)  127 (35.0)  96 (26.5)  38 (10.5) | p=0.943 ^c^ | |
| Obesity  BMI <30  BMI ≥ 30 | 286 (44.1)  362 (55.9) | 187 (51.5)  176 (48.5) | p=0.024 ^b^ | |
| Systolic blood pressure (mmHg), mean (SD) | 135.7 (17.9) | 134.5 (19.2) | p=0.342 ^a^ | |
| Change in systolic blood pressure, mean (SD) | 2.2 (14.0) | N/A | N/A | |
| Diastolic blood pressure (mmHg), mean (SD) | 86.4 (9.9) | 86.1 (10.2) | p=0.692 ^a^ | |
| Change in diastolic blood pressure, mean (SD) | 0.4 (7.8) | N/A | N/A | |
| Hypertension, freq (%)  Never diagnosed  Diagnosed | 246 (38.0)  402 (62.0) | 138 (38.0)  225 (62.0) | p=0.987 ^b^ | |
| Antihypertensive treatment, freq (%)  Not taking  Taking | 359 (55.4)  289 (44.6) | 207 (57.0)  156 (43.0) | p=0.618 ^b^ | |
| Depressive symptoms at baseline  Normal-mild (DASS <14)  Moderate to very severe (DASS≥14) | 575 (88.7)  73 (11.3) | 307 (85.5)  52 (14.5) | p=0.138 ^b^ | |

*mmHg: millimetres of mercury, DASS: Depression Anxiety Stress Scale, BMI: body mass index, SD: standard deviation, IQR: interquartile range. Normal to mild depressive symptoms if depression subscale of 21-item Depression Anxiety Stress Scale score was <14. Moderate to very severe depressive symptoms if depression subscale of 21-item Depression Anxiety Stress Scale score was ≥14. ^a^t-test; ^b^Chi^2^ test; ^c^Kruskall-Wallis test*
