# Supplementary material for: Prospective association between depressive symptoms and blood-pressure related outcomes in Kosovo
Source: PLOS Glob Public Health. 2023 Apr 7;3(4):e0000851. doi: 10.1371/journal.pgph.0000851 (PMC10081745; doi:10.1371/journal.pgph.0000851)
Supplement: S2 Table — Results from multivariable censored regression models. a Minimally adjusted: Age (in years), sex (male, female), highest level of education completed (primary school or less, secondary school, university/college or more), work (working, home-person, retired/disabled, unemployed), urban-rural classification (rural, urban), ethnicity (Albanian, Serbian, Roma/Ashkali/Egyptian/Other). b Fully adjusted: Minimally adjusted covariates and additionally, smoking status (current smoker), physical inactivity (<150 min of moderate-intensity physical activity per week, or <75 min of vigorous-intensity physical activity per week, or less than an equivalent combination of moderate-intensity. DASS-21: 21-item Depression Anxiety Stress Scale, Ref: Reference group. (DOCX) [file pgph.0000851.s002.docx]

**S2 Table**. Prospective association between depression and change in systolic and diastolic blood pressure per year, without adjustment for baseline systolic and diastolic blood pressure

|  | **Change in systolic blood pressure** | | | | | | | **Change in diastolic blood pressure** | | | | | |  |
| --- | --- | --- | --- | --- | --- | --- | --- | --- | --- | --- | --- | --- | --- | --- |
|  | Minimally ^a^ adjusted | | | Fully ^b^ adjusted | | | | Minimally ^a^ adjusted | | | Fully ^b^ adjusted | | | |
|  | Coef | 95%-CI | p-value | Coef | 95%-CI | p-value | | Coef | 95%-CI | p-value | Coef | 95%-CI | p-value | |
| **Depression**  Normal to mild depressive symptoms (DASS<14)  Moderate to very severe depressive symptoms (DASS ≥14) | (Ref)  -2.64 | (-6.32, 1.03) | p=0.155 | (Ref)  -2.27 | (-6.04, 1.50) | | p=0.238 | (Ref)  -3.48 | (-5.48, -1.49) | p=0.001 | (Ref)  -3.23 | (-5.26, -1.19) | p=0.002 | |

| **Depression severity (categorical)**  Normal (DASS-21 score 0-9)  Mild (DASS-21 score 10-13)  Moderate (DASS-21 score 14-20)  Severe (DASS-21 score 21-27)  Very severe (DASS-21 score ≥ 28) | (Ref)  -0.63  -4.14  -1.47  2.30 | (-4.93, 3.67)  (-8.49, 0.21)  (-9.92, 6.99)  (-6.33, 10.94) | p=0.773  p=0.062  p=0.733  p=0.601 | (Ref)  -0.24  -3.95  -0.19  2.99 | (-4.58, 4.09)  (-8.37, 0.46)  (-8.64, 8.27)  (-5.74, 11.71) | p=0.913  p=0.079  p=0.966  p=0.503 | (Ref)  0.93  -4.09  -2.55  -1.01 | (-1.36, 3.22)  (-6.43, -1.75)  (-7.10, 1.99)  (-5.62, 3.60) | p=0.427  p=0.001  p=0.271  p=0.677 | (Ref)  1.19  -3.93  -2.07  -0.12 | (-1.12, 3.49)  (-6.31, -1.56)  (-6.61, 2.48)  (-4.79, 4.54) | p=0.314  p=0.001  p=0.373  p=0.958 |
| --- | --- | --- | --- | --- | --- | --- | --- | --- | --- | --- | --- | --- |

*Results from multivariable censored regression models. ^a^ minimally adjusted: age (in years), sex (male, female), highest level of education completed (primary school or less, secondary school, university/college or more), work (working, home-person, retired/disabled, unemployed), urban-rural classification (rural, urban), ethnicity (Albanian, Serbian, Roma/Ashkali/Egyptian/Other). ^b^ fully adjusted: minimally adjusted covariates and additionally, smoking status (current smoker), physical inactivity (<150 min of moderate-intensity physical activity per week, or <75 min of vigorous-intensity physical activity per week, or less than an equivalent combination of moderate-intensity and vigorous-intensity activity; poor nutrition (<5 fruits and/or vegetables per day), alcohol consumption (any alcohol in the last 30 days), obesity (BMI≥30), heart rate (beats per minutes), number of main family medicine center visits in the last 6 months. DASS-21: 21-item Depression Anxiety Stress Scale; Ref: Reference group; mmHg: millimetres of mercury*
