# Supplementary material for: Prospective association between depressive symptoms and blood-pressure related outcomes in Kosovo
Source: PLOS Glob Public Health. 2023 Apr 7;3(4):e0000851. doi: 10.1371/journal.pgph.0000851 (PMC10081745; doi:10.1371/journal.pgph.0000851)
Supplement: S3 Table — (DOCX) [file pgph.0000851.s003.docx]

**S3 Table.** Prospective association between depression and change in systolic and diastolic blood pressure, minimally adjusted model and introduction of potential mediators individually

|  | | **Change in systolic blood pressure** | | | **Change in diastolic blood pressure** | | |
| --- | --- | --- | --- | --- | --- | --- | --- |
|  |  | Coef | 95%-CI | p-value | Coef | 95%-CI | p-value |
| Minimally^a^ adjusted | **Depression**  Normal to mild depressive symptoms (DASS<14)  Moderate to very severe depressive symptoms (DASS ≥14) | (Ref)  -2.31 | (-5.48, 0.87) | p=0.155 | (Ref)  -2.93 | (-4.68, -1.18) | p=0.001 |
| Minimally^a^ adjusted + adjustment for **physical inactivity** | **Depression**  Normal to mild depressive symptoms (DASS<14)  Moderate to very severe depressive symptoms (DASS ≥14) | (Ref)  -2.30 | (-5.48, 0.87) | p=0.155 | (Ref)  -2.93 | (4.68, -1.17) | p=0.001 |
| Minimally^a^ adjusted + adjustment for **smoking** | **Depression**  Normal to mild depressive symptoms (DASS<14)  Moderate to very severe depressive symptoms (DASS ≥14) | (Ref)  -2.44 | (-5.62, 0.75) | p=0.134 | (Ref)  -3.02 | (-4.78, -1.25) | p=0.001 |
| Minimally^a^ adjusted + adjustment for **alcohol** | **Depression**  Normal to mild depressive symptoms (DASS<14)  Moderate to very severe depressive symptoms (DASS ≥14) | (Ref)  -2.27 | (-5.45, 0.91) | p=0.161 | (Ref)  -2.91 | (-4.66, -1.16) | p=0.001 |
| Minimally^a^ adjusted + adjustment for **poor nutrition** | **Depression**  Normal to mild depressive symptoms (DASS<14)  Moderate to very severe depressive symptoms (DASS ≥14) | (Ref)  -2.25 | (-5.43, 0.94) | p=0.166 | (Ref)  -2.85 | (-4.60, -1.09) | p=0.001 |
| Minimally^a^ adjusted + adjustment for **sleep quality** | **Depression**  Normal to mild depressive symptoms (DASS<14)  Moderate to very severe depressive symptoms (DASS ≥14) | (Ref)  -2.04 | (-5.28, 1.21) | p=0.219 | (Ref)  -2.81 | (-4.61, -1.01) | p=0.002 |
| Minimally^a^ adjusted + adjustment for **obesity** | **Depression**  Normal to mild depressive symptoms (DASS<14)  Moderate to very severe depressive symptoms (DASS ≥14) | (Ref)  -2.29 | (-5.46, 0.89) | p=0.158 | (Ref)  -2.93 | (-4.69, -1.16) | p=0.001 |
| Minimally^a^ adjusted + adjustment for **heart rate** | **Depression**  Normal to mild depressive symptoms (DASS<14)  Moderate to very severe depressive symptoms (DASS ≥14) | (Ref)  -2.43 | (-5.60, 0.74) | p=0.133 | (Ref)  -3.05 | (-4.80, -1.31) | p=0.001 |
| Minimally^a^ adjusted + adjustment for **visits to Main Family Medicine Centers** | **Depression**  Normal to mild depressive symptoms (DASS<14)  Moderate to very severe depressive symptoms (DASS ≥14) | (Ref)  -2.13 | (-5.31, 1.05) | p=0.188 | (Ref)  -2.87 | (-4.64, -1.10) | p=0.002 |

*Smoking status (current smoker), physical inactivity (<150 min of moderate-intensity physical activity per week, or <75 min of vigorous-intensity physical activity per week, or less than an equivalent combination of moderate-intensity and vigorous-intensity activity; poor nutrition (<5 fruits and/or vegetables per day), alcohol consumption (any alcohol in the last 30 days), obesity (BMI≥30), heart rate (beats per minutes), number of main family medicine center visits in the last 6 months. DASS-21: 21-item Depression Anxiety Stress Scale*
