# Supplementary material for: Prospective association between depressive symptoms and blood-pressure related outcomes in Kosovo
Source: PLOS Glob Public Health. 2023 Apr 7;3(4):e0000851. doi: 10.1371/journal.pgph.0000851 (PMC10081745; doi:10.1371/journal.pgph.0000851)
Supplement: S4 Table — (DOCX) [file pgph.0000851.s004.docx]

**S4 Table.** P-values of the interaction term between depression and sex in main fully adjusted models

|  | p-value of interaction term between depression and sex |
| --- | --- |
| Prospective association between depression and change in systolic blood pressure | p=0.827 |
| Prospective association between depression and change in diastolic blood pressure | p=0.977 |
| Prospective association between depression and hypertension diagnosis | p=0.354 |
| Prospective association between depression and uncontrolled hypertension | p=0.919 |
