# Supplementary material for: Prospective association between depressive symptoms and blood-pressure related outcomes in Kosovo
Source: PLOS Glob Public Health. 2023 Apr 7;3(4):e0000851. doi: 10.1371/journal.pgph.0000851 (PMC10081745; doi:10.1371/journal.pgph.0000851)
Supplement: Attachment — Submitted filename: Response to Reviewers #2.docx [file pgph.0000851.s005.docx]

Anil Gumber, Ph.D.
Academic Editor
PLOS Global Public Health

Basel, 15.07.2022

Response to Reviewers #2, Re: original research article titled: “Prospective association between depressive symptoms and blood-pressure related outcomes in Kosovo”

Dear Dr. Anil Gumber,

In response to your request for further information in regards to our request for an exception to public deposition of the data, please see the contact persons below:

**Ethics committee imposing restrictions:** Ethikkommission Nordwest- und Zentralschweiz (Ref. 2018-00994)

**Data access committee:**

1. **Prof. Dr. Nicole Probst-Hensch**

Head of Department, Epidemiology and Public Health

PI of KOSCO study

Swiss Tropical and Public Health Institute

nicole.probst@swisstph.ch

Direct +41 61 284 83 78

Mobile +41 79 280 34 14

2. **Dr. Malin Ziehmer-Wenz**

Chief Information Security and Data Privacy Officer

Swiss Tropical and Public Health Institute

[malin.ziehmer-wenz@swisstph.ch](javascript:linkTo_UnCryptMailto('nbjmup+nbmjo\/ajfinfs.xfoaAtxjttuqi\/di');)
Direct [+41 61 284 87 98](tel:+41612848798)

We continue to believe that our research article is of interest to your readership and valuable to the scientific community. We hope that you will consider our research for publication and look forward to your response to our resubmission.

Kind regards,

Nicole Probst-Hensch

*Professor, Dr. phil. et PhD*

*Head of Department,*

*Epidemiology and Public Health*

*Swiss Tropical and Public Health Institute*

Authors:

**Katrina A. Obas, PhD**
katrina.obas@swisstph.ch, 1) Department of Epidemiology and Public Health, Swiss Tropical and Public Health Institute, Socinstrasse 57, Basel 4002, Switzerland, 2) University of Basel, Petersplatz 1, 4001, Basel, Switzerland

**Marek Kwiatkowski, PhD**
marek.kwiatkowski@swisstph.ch 1) Department of Epidemiology and Public Health, Swiss Tropical and Public Health Institute, Socinstrasse 57, Basel 4002, Switzerland, 2) University of Basel, Petersplatz 1, 4001, Basel, Switzerland

**Ariana Bytyci-Katanolli**
ariana.bytyci@swisstph.ch, 1) Department of Epidemiology and Public Health, Swiss Tropical and Public Health Institute, Socinstrasse 57, Basel 4002, Switzerland, 2) University of Basel, Petersplatz 1, 4001, Basel, Switzerland

**Shurkije Statovci, MD**

shukistatovci@gmail.com, 1) University Clinical Center of Kosovo, 10000, Prishtina

**Naim Jerliu, Professor, PhD**

jerliui@gmail.com, 1) National Institute of Public Health, Str. Instituti Shendetesor, NN, 10000, Prishtina, Kosovo, 2) University of Prishtina Medical Faculty, Str. Bulevardi i Deshmoreve, NN, 10 000 Prishtina, Kosovo

**Qamile Ramadani, MD**

qamile.ramadani@aqhproject.org, 1) Accessible Quality Healthcare Project Implementation Unit, Tirana str. Prime Residence, C 4/3 Entrance D, Floor 6, Flats no. 16, 17 & 18, 10000, Prishtina, Kosovo.

**Nicu Fota**

nicu.fota@aqhproject.org, 1) Accessible Quality Healthcare Project Implementation Unit, Tirana str. Prime Residence, C 4/3 Entrance D, Floor 6, Flats no. 16, 17 & 18, 10000, Prishtina, Kosovo.

**Jana Gerold, PhD**
jana.gerold@swisstph.ch, 1) Swiss Centre for International Health, Swiss Tropical and Public Health Institute, Kreuzstrasse 2, 4123 Allschwill, Switzerland, 2) University of Basel, Petersplatz 1, 4001, Basel, Switzerland

**Manfred Zahorka, MD**
m.zahorka@optimedis.de, 1) Swiss Centre for International Health, Swiss Tropical and Public Health Institute, Kreuzstrasse 2, 4123 Allschwill, Switzerland, 2) University of Basel, Petersplatz 1, 4001, Basel, Switzerland

**Nicole Probst-Hensch, Professor, PhD, MPH, corresponding author**
nicole.probst@swisstph.ch, 1) Department of Epidemiology and Public Health, Swiss Tropical and Public Health Institute, Socinstrasse 57, Basel 4002, Switzerland, 2) University of Basel, Petersplatz 1, 4001, Basel, Switzerland
