# Supplementary material for: Prospective association between depressive symptoms and blood-pressure related outcomes in Kosovo
Source: PLOS Glob Public Health. 2023 Apr 7;3(4):e0000851. doi: 10.1371/journal.pgph.0000851 (PMC10081745; doi:10.1371/journal.pgph.0000851)
Supplement: Attachment — Submitted filename: Response to Reviewers.docx [file pgph.0000851.s006.docx]

Steve Zimmerman, PhD
PLOS Staff Editor
PLOS Global Public Health

Basel, 14.12.2022

Response to Reviewers, Re: original research article titled: “Prospective association between depressive symptoms and blood-pressure related outcomes in Kosovo”

Dear Dr. Steve Zimmerman,

We appreciate the time and effort that you and the reviewers have dedicated to providing feedback on our manuscript and are grateful for the insightful comments and valuable improvements to our paper. We have incorporated most of the suggestions made by the reviewers. Those changes are marked up within the manuscript with “track changes”. Please see below, in blue, for point-by-point response to the reviewers’ comments and concerns. All page numbers refer to the revised manuscript file with track changes.

We continue to believe that our research article is of interest to your readership and valuable to the scientific community. We hope that you will consider our research for publication and look forward to your response to our resubmission.

Kind regards,

Nicole Probst-Hensch

*Professor, Dr. phil. et PhD*

*Head of Department,*

*Epidemiology and Public Health*

*Swiss Tropical and Public Health Institute*

**Journal Requirements:**

1. Please review your reference list to ensure that it is complete and correct. If you have cited papers that have been retracted, please include the rationale for doing so in the manuscript text, or remove these references and replace them with relevant current references. Any changes to the reference list should be mentioned in the rebuttal letter that accompanies your revised manuscript. If you need to cite a retracted article, indicate the article’s retracted status in the References list and also include a citation and full reference for the retraction notice.

The reference list (starting on page 35) has been verified to comply with the Vancouver style. We have updated the two references (originally numbered 3 and 14), which were in press at the time of submission but are now published, to the full publication reference. We have also added references in response to the reviewer’s request to expand on the discussion.

2. In the online submission form, you indicated that "The datasets generated and analyzed during the current study are not publicly available due to non-anonymized data in the context of cohort data but are available on reasonable request". All PLOS journals now require all data underlying the findings described in their manuscript to be freely available to other researchers, either 1. In a public repository, 2. Within the manuscript itself, or 3. Uploaded as supplementary information. This policy applies to all data except where public deposition would breach compliance with the protocol approved by your research ethics board. If your data cannot be made publicly available for ethical or legal reasons (e.g., public availability would compromise patient privacy), please explain your reasons by return email and your exemption request will be escalated to the editor for approval. Your exemption request will be handled independently and will not hold up the peer review process, but will need to be resolved should your manuscript be accepted for publication. One of the Editorial team will then be in touch if there are any issues.

Our dataset cannot be made publicly available for ethical reasons. We have submitted a follow-up letter to the editor concerning our exemption request since we have already submitted a request and follow-up documentation. Public deposition of data would breach compliance with the protocol approved by our ethics board in Switzerland and in Kosovo.

Additional Editor Comments (if provided):
[Note: HTML markup is below. Please do not edit.]
Reviewers' comments:
Reviewer's Responses to Questions

**Comments to the Author**

1. If the authors have adequately addressed your comments raised in a previous round of review and you feel that this manuscript is now acceptable for publication, you may indicate that here to bypass the “Comments to the Author” section, enter your conflict of interest statement in the “Confidential to Editor” section, and submit your "Accept" recommendation.

Reviewer #1: (No Response)

Reviewer #2: All comments have been addressed

Reviewer #3: (No Response)

2. Does this manuscript meet PLOS Global Public Health’s [publication criteria](https://journals.plos.org/globalpublichealth/s/criteria-for-publication)? Is the manuscript technically sound, and do the data support the conclusions? The manuscript must describe methodologically and ethically rigorous research with conclusions that are appropriately drawn based on the data presented.

Reviewer #1: Partly

Reviewer #2: Yes

Reviewer #3: Partly

3. Has the statistical analysis been performed appropriately and rigorously?

Reviewer #1: Yes

Reviewer #2: Yes

Reviewer #3: Yes

4. Have the authors made all data underlying the findings in their manuscript fully available (please refer to the Data Availability Statement at the start of the manuscript PDF file)?

The [PLOS Data policy](https://journals.plos.org/globalpublichealth/s/data-availability) requires authors to make all data underlying the findings described in their manuscript fully available without restriction, with rare exception. The data should be provided as part of the manuscript or its supporting information, or deposited to a public repository. For example, in addition to summary statistics, the data points behind means, medians and variance measures should be available. If there are restrictions on publicly sharing data—e.g. participant privacy or use of data from a third party—those must be specified.

Reviewer #1: No

Reviewer #2: Yes

Reviewer #3: No

5. Is the manuscript presented in an intelligible fashion and written in standard English?

PLOS Global Public Health does not copyedit accepted manuscripts, so the language in submitted articles must be clear, correct, and unambiguous. Any typographical or grammatical errors should be corrected at revision, so please note any specific errors here.

Reviewer #1: Yes

Reviewer #2: Yes

Reviewer #3: Yes

6. Review Comments to the Author

Please use the space provided to explain your answers to the questions above. You may also include additional comments for the author, including concerns about dual publication, research ethics, or publication ethics. (Please upload your review as an attachment if it exceeds 20,000 characters)

**Reviewer #1:**

Thank you for submitting your work on depressive symptomatology and hypertension. I read your work with great interest, as these are areas of growing concern globally, and as your introduction demonstrates, are issues of high concern in Kosovo. With revisions to the work, I’m sure there are many academics, and primary care and public health practitioners, who would find your study to be of great importance.

Thank you very much for this positive feedback.

Overall, I feel the introduction, methods, and results are done to a high quality and are reflective of the care and consideration put into the study. Unfortunately, I feel there are several areas of the discussion section that need further revision, and additionally there are missing areas of the discussion section which I feel would be of importance to the reader. I appreciate a discussion section can be more difficult when the majority of findings are negative or non-significant, however, as you surely agree negative findings are of equal importance to the research community and I’m sure with revision of the discussion section there is much we can learn from your results.

Thank you for the positive feedback in regards to the introduction, methods and results as well as the constructive feedback for our discussion, which contribute to strengthening our study.

My main concern with the discussion section is that it presents a single explanation (with regards to associations with people with depressive symptoms) for the paper’s findings regarding hypertension diagnosis (increased healthcare utilisation), and a single explanation for attenuated blood pressure (monoamine theory of depression). I feel both explanations are speculative, and although they are explained, they receive minimal evidence or justification from either the study’s findings, or other literature. There are a huge range of possible explanations and confounding factors for why depressive symptoms may be associated with slightly lower blood pressure in the first year of diagnosis, and why there may be an association with different rates of diagnosis and control of blood pressure. Broadly these could be biological, psychological, social and healthcare types of factors, and within each category there could be many explanations or confounding factors. There may be a variety of factors at play rather than a single explanation. It is also notable as well that the authors single out their inclusion of healthcare utilisation as a covariate as a possible reason for the non-significance of several findings, but do not grant this privilege to any other covariates (could smoking or physical inactivity equally exist along the causal pathway?). It will likely be unclear to the reader why the reasons given have been singled out, and a broader discussion would be of greater interest. In general, I would urge caution with some of the suggestions or conclusions proposed in the discussion, many of which are purely speculative and cannot be adequately justified by the results.

Thank you for the careful evaluation of our discussion section. We agree with this comment and have adapted the discussion on pages 25-31 to include a wider scope of possible explanations for the findings with supporting evidence from the literature, also explained following your related comment below. We specifically explain that we aimed to replicate the findings of our Swiss study. We added a sentence in the conclusion section stating that future studies need to address the full complexity of potential pathways.

Additionally, the discussion section would benefit from suggesting implications for clinicians or policymakers. There is reference to the work being of epidemiological interest, and in the conclusion section, of the need for larger studies. Please include discussion of what implications there are for the work for primary care providers and for those in public health. Additionally, other than conducting larger studies, how else should further research in this area be directed based on the experience of this study?

We have included a paragraph on implications for clinicians and policy makers on pages 29-30. We added a sentence the conclusion section stating that future studies need to address the full complexity of potential pathways.

Some of the methods could be enhanced by further discussion or explanation to the reader. I wasn’t personally familiar with the method used for censored normal regression in blood pressure follow-up over time, and it would be useful to explain why this was used and what effect it had on the results. Use of censored regressions does likely effect how the results should be interpreted. The models also include a relatively high number of covariates, and there is likely overlap between many of the covariates (e.g. alcohol consumption and nutrition). Could this have affected the ability of the regression models to find significant changes?

We understand that the use of censored regression to assess the outcome of blood pressure is a novel method and likely not well known among most readers. However, we believe that we explained the method in a clear and consice way, and that the essential aspects for interpretation of our findings are included in the manuscript. We think that going into further detail about censored regression in the manuscript could bring the reader away from the focus of the paper. In case the reader is interested in understanding censored regression in greater detail, it can be retrieved from the reference we included.

Specifically, we describe our motivation to use censored regression (why it is used) in the first paragraph of the statistical methods, on page 15: “Many study participants who were normotensive at baseline developed hypertension and were prescribed antihypertensive medication before their follow-up health assessments. Other participants who took antihypertensive medication at baseline stopped treatment by follow-up 3 for various reasons. Accounting for the effect of this treatment on the measured blood pressure is of vital importance in statistical analyses of blood pressure.”

This section also explains which values are censored and how censoring should be interpreted: “In this approach, the blood pressure change for all participants newly diagnosed or treated for hypertension at follow-up 3 was right-censored at the measured value. This is equivalent to assuming that had these participants not received the diagnosis, their blood pressure at the follow-up assessment would have been equal to or greater than the measured value.” Also on page 13 “A positive censored regression coefficient for moderate to very severe depressive symptoms indicates larger increase in blood pressure over time compared to the reference group, while a negative coefficient represents a smaller increase in blood pressure over time compared to the reference group. A negative coefficient should not be interpreted as a decrease in blood pressure over time.”

In regards to the number of covariates included in our models: Our intention was to understand the effect of depressive symptoms on blood pressure change beyond the mediating effect from lifestyle, given that it is well established that lifestyle factors exist along the causal pathway between depression and hypertension. We therefore believe that our covariate selection is appropriate after careful consideration of the literature. Further, we do not believe alcohol and nutrition overlap in this population, as we have observed in a previous study from this population that alcohol consumption and poor nutrition have strongly differing frequencies, which are specific to this cultural context.

Finally, the section on weaknesses/limitations omits possible concerns about the study which should at the very least be stated. One example would be around how the study population may not represent a general population, and therefore may mean the results are not fully generalisable. For instance, there are several areas of possible selection bias in the study population: firstly, the vast majority of those eligible declined to participate; secondly, a large proportion were lost to follow-up; thirdly, within the paper it is not stated where or how patients were approached for recruitment (although reference is made to a study protocol elsewhere). It was not clear to me if the study itself also affected the rate at which people attended their primary care provider. For instance, how linked were the study visits to the primary care provided (either in selection or follow-up), and what happened if a participant had a high blood pressure reading detected in a study follow-up which was not previously known about? Additionally, this would be a good place to discuss the limitations of the study size, and the length of follow-up, which are alluded to elsewhere in the paper.

We have elaborated on the limitations of the study in relation to selection bias on pages 30-31. Length of follow-up was already mentioned as a limitation in this section.

We have also added details of the recruitment method, the link between the study and healthcare services page 6 and 8. The protocol to disclose high blood pressure readings to patients has been added to page 13.

I additionally note that the data is not routinely made publicly available, although the appropriateness of this matter would be better addressed by the editor.

We have addressed this concern with the editor.

Line 53: Please be more precise than this in describing the evidence for depression and CVD as not all readers may be familiar with it, and it is of very high importance to your study. It would be useful to state briefly what the proposed relationship between depression and CVD is (e.g., if it’s a risk factor and in what direction), and if this has been demonstrated at the level of primary studies, or meta-analyses etc. This would aid readers in understanding the high importance of your research.

We have now clarified the direction the of association (depression independent risk factor for CVD), the type of study (meta-analyses) and effect sizes /confidence intervals on page 4.

Lines 60-61: Whilst generally accepted that hypertension is an important risk factor for CVD, I feel you should include a reference if stating it is the most important of all risk factors.

We have included two references which support the claim that hypertension is a main risk factor for CVD as well as in general on page 4.

Line 83: It seems significant that the study was conducted in primary care centres that do not offer mental health services. In your introduction (lines 67-69) you reference work which suggested that increased healthcare utilisation by those seeking help for depression might lead to higher hypertension diagnosis. Could this aspect of the primary care centres have affected your results in anyway? I could not see this referenced to in the discussion section.

The sentence in lines 67-69 may have been misinterpreted. Other studies have observed higher healthcare utilization among people with depressive symptomology compared to those without, regardless of the reason for seeking healthcare. Therefore we did not intend to indicate that the reason for healthcare seeking was related to their mental health needs. The wording has now been clarified on page 5.

We would also like to add that we did indeed consider that perhaps the increased healthcare utilization observed among depressed people would influence our outcome of hypertension if the outcome definition included hypertension diagnosis, and this is precisely the argument why we chose to use change in blood pressure as a separate outcome. We have added this argumentation in the introduction on page 5.

Lines 96 & 98: It is not fully clear what is meant by ‘follow-up 3,’ is this meant to be the third follow-up visit? It’s not stated elsewhere how many follow-up visits occur or in what timeframe they occur. It may be worth rewording this, and further explaining the timeline of follow-up.

Thank you for pointing to this oversight on our part. The timeline of the study is now described with a short text and table on page 7.

Lines 176 & 179: Both lines feature clauses in brackets, which are missing closed brackets.

The closed brackets have now beed added on page 16.

Line 184: Please remove ‘a’ as you are referring to the plural form of multiple logistic regression models.

The ‘a’ has been removed on page 16.

Line 202 & 230-233: Whilst you make good points here, commenting on the significance of your results, and comparison to other literature, would be better suited to the discussion section.

Lines 230-233 were moved from the discussion section on pages 27-28.

Line 242-248: There are many possible explanations for why those with moderate-to-severe depressive symptomatology may have an association with higher rates of hypertension diagnosis, and/or blood pressure attenuation. Your research has observed (mostly non-significant) tendencies but it has not investigated the reasons behind them. I think it is premature to single-out explanations, especially without reference to supporting literature or discussion of other plausible mechanisms. It is also not clear if your research findings definitively support any of the proposed explanations or why else they have been selected. It was also not clear from the paper how patients were recruited, and if those who were recruited had a higher tendency to healthcare-seeking than a baseline population of depressed patients (e.g. were patients recruited in a primary care centre they were already choosing to attend?). I don’t feel the study as it’s currently explained supports the suggestion that healthcare utilisation is primarily responsible for increased diagnosis. Furthermore, many of the findings were non-significant, which you only state by implication (‘only the findings with attenuated diastolic blood pressure met statistical significance’), instead of making this more explicit.

As already mentioned above at your first comment in regards to limited explanations explored for the findings: We agree with this comment and have adapted the discussion on pages 25-31 to include a wider scope of possible explanations for the findings with supporting evidence from the literature.

Also mentioned previously, we have added details of the recruitment method and the link between the study and healthcare visits on pages 6 and 8.

Lines 252-257: There is a huge variety of proposed theories and mechanisms for depression, and neurochemical explanations in particular are currently under quite intense scrutiny. If you wish to discuss this in relation to your hypertension findings, I feel it requires further explanation and justification to the reader. Or alternatively, making more explicit that there is a much wider field of possible explanations for the pathogenesis of depression (some of which may also have overlap with explanations for hypertension).

Further explanation to the neurochemical theories were added on page 26.

Lines 258-260: It is very interesting that there was minimal discrepancy between your change in systolic and diastolic blood pressure findings, between minimally- and fully-adjusted models. Perhaps this reflects relationships between your ‘minimal’ covariates (e.g. gender, educational attainment) and your ‘maximal’ covariates (e.g. smoking, physical activity).

Thank you for this observation. We have elaborated on the potential relationship between minimal and maximal covariates on page 27.

Lines 271-275: It might be helpful to further justify this within the context of primary care in Kosovo. For instance, is it standard practice in Kosovo to routinely assess patients with depression for hypertension? This may not be standard practice globally in routine mental health primary care visits, and it would be useful to highlight its practical importance to clinicians.

A line has been added to specify mental health care standards in Kosovo on page 8.

Lines 266-268: ‘We found suggestive evidence that initially normotensive people with depressive symptoms were more likely to have hypertension detected by a general practitioner if it developed in comparison to non-depressed’. I think it would be best to omit ‘if it developed,’ as I don’t believe your study comparatively reported new hypertension which was undiagnosed in primary care (did your study follow-ups detect any patients with hypertension that had been missed by their primary care visits?). It would be more accurate just to state that normotensive people with depressive symptoms were more likely to have had hypertension diagnosed later on in follow-up. It would be interesting to know if there was a higher rate of undiagnosed hypertension in either group.

We have omitted ‘if it developed’ on page 27.

Lines 273-275: This is an excellent point, however you do include many covariates in your models, all of which could contain partial confounding factors or explanations for the relationship between depression symptoms and hypertension diagnosis/control. As before, it is not fully clear to me your justification for only choosing to discuss healthcare utilisation.

We focus on healthcare utilization to potentially explain our findings because it is well established that lifestyle factors exist on the causal pathway between depression and hypertension. Healthcare utilization has not yet been explored as a reason to explain the association.

Lines 276-286: I would omit this paragraph. In proposing psychosomatic complaints as a basis for your findings, you are asking the reader to accept many steps, namely that i) those with depression attend their primary care provider more frequently; ii) a significant proportion or majority of these visits are for physical complaints and not depression; iii) the majority of these physical complaints are psychosomatic in nature; iv) all patients presenting with psychosomatic complaints have a blood pressure reading taken; v) any blood pressure reading beyond 140/90mmHg is treated as a separate issue and triggers a diagnostic pathway for hypertension. Furthermore, you support this only with a single reference to an observational study. Your final sentence, is a simplified statement on a very complex issue in primary care (and made with reference to an article from 1990; great strides have been made in primary care in the intervening decades in understanding psychosomatic illness and mental health). It’s quite a significant leap in my view to start proposing this a reason for your findings.

The paragraph on page 28-29 has been omitted as we agree that the explanation provided makes several assumptions.

Lines 288-299: To refine this paragraphs readability, please revise the use of the word ‘however’ at the beginning of 3 different sentences.

The redundant “however” was changed to “nevertheless” on page 29.

Lines 292-294: You make a very interesting point here regarding the relationship between ethnic groups and hypertension control. I note that there is a significantly higher proportion of people of Roma, Ashkali, Egyptian, and other ethnicities represented in your group with moderate to severe depression symptoms. I’m sure this would be of great interest to the reader, particularly given that it is more unique to the setting of Kosovo. Could you expand further on this point, or is there other literature from Kosovo that may further expand this point?

Thank you for this input. Due to ethnically-related conflicts during the war in the country, reporting ethnicity was forbidden in data collection to protect its citizens from persecution until recently and only for specific reasons with ethical approval could ethnicity be collected. Therefore the evidence on cultural differences in health related outcomes are not available in the country. We have however added reference to other studies about minorities on page 29.

Lines 310-311: I think you should make clear in the methods section that there was this degree of loss to follow-up as it does affect the reader’s interpretation of your study. Furthermore, you should state in the methods section how you handled subjects lost to follow-up.

We have moved the paragraph on loss to follow-up in the discussion / limitations section to the methods section on page 9 as the reviewer recommends. In this paragraph, it also included a description how there were no differences in characteristics between those lost to follow-up and those included in the study, which are included in the Additional file 1 (previously 3). A short summary sentence has taken its place in the discussion / limitations section.

Lines 312-314: It is interesting that you found many of those lost to follow-up, stopped attending due to Covid. Could this of affected the selection of your patient population, for instance were only healthier or younger participants more likely to accept the risk of coming for follow-up? Could this population of had a difference in the sequelae of a hypertension diagnosis?

The observation that participants refrained from participating in follow-ups during covid was from verbal responses when study nurses called participants to organize a meeting for follow-up. We have nevertheless compared participants and those loss to follow-up as mentioned in the previous comment. We have added a sentence to the limitations of the study.

Line 317-319: Firstly, I think the conclusion about age-related diastolic blood pressure change should be further clarified as something only detected within around a year of follow-up. Secondly, even though you state it is only ‘suggestive,’ I don’t think it would be appropriate to include the findings on detection and hypertension control as conclusions, given they were non-significant.

We have removed the wording “age-related” blood pressure change throughout the manuscript given as you mention that the change is only observed over a year.

Lines 318-320: This is far too general a statement, I don’t think your study demonstrated definitively that there was no relationship between depression, hypertension, and CVD risk. I would omit reference to CVD risk in your conclusion, as your study doesn’t directly investigate incidence or risk of CVD.

We have reformulated the sentence on CVD risk in our conclusion on page 31.

Reviewer #2: (No Response)

Reviewer #3: ..

7. PLOS authors have the option to publish the peer review history of their article ([what does this mean?](https://journals.plos.org/globalpublichealth/s/editorial-and-peer-review-process#loc-peer-review-history)). If published, this will include your full peer review and any attached files.

**Do you want your identity to be public for this peer review?** If you choose “no”, your identity will remain anonymous but your review may still be made public.

For information about this choice, including consent withdrawal, please see our [Privacy Policy](https://www.plos.org/privacy-policy).

Reviewer #1: No

Reviewer #2: No

Reviewer #3: No

[NOTE: If reviewer comments were submitted as an attachment file, they will be attached to this email and accessible via the submission site. Please log into your account, locate the manuscript record, and check for the action link "View Attachments". If this link does not appear, there are no attachment files.]

While revising your submission, please upload your figure files to the Preflight Analysis and Conversion Engine (PACE) digital diagnostic tool, https://pacev2.apexcovantage.com/. PACE helps ensure that figures meet PLOS requirements. To use PACE, you must first register as a user. Registration is free. Then, login and navigate to the UPLOAD tab, where you will find detailed instructions on how to use the tool. If you encounter any issues or have any questions when using PACE, please email PLOS at figures@plos.org. Please note that Supporting Information files do not need this step.
