# Supplementary material for: Prospective association between depressive symptoms and blood-pressure related outcomes in Kosovo
Source: PLOS Glob Public Health. 2023 Apr 7;3(4):e0000851. doi: 10.1371/journal.pgph.0000851 (PMC10081745; doi:10.1371/journal.pgph.0000851)
Supplement: Attachment — Submitted filename: 20230208 Response to Reviewers.docx [file pgph.0000851.s007.docx]

**Reviewer 2 comments**

Thank you for inviting me to re-review this study entitled "Prospective association between depressive symptoms and blood-pressure related outcomes in Kosovo."

We appreciate the time and effort that you have dedicated to providing feedback on our manuscript and are grateful for the insightful comments and valuable improvements to our paper.

**General comment:** I appreciate the authors' efforts to address all comments well. If possible, the author also should include some studies conducted in developing countries like Asia, Africa and developed countries to enrich this manuscript. However, I request the authors to consider the following suggestions for further improvement of the manuscript.

Thank you for this suggestion. We have added studies about prevalence of depression in Asia and Africa during the COVID-19 pandemic to put the prevalence in Kosovo into perspective among other LMIICs.

1. The references are also quite old more than 10 years accounting for about 45% of the reference list, and should not exceed more than 20%.

We attribute the higher proportion of references before 2013 to the following:

1) We criticize the last meta-analysis (to the best of our knowledge) on the subject of prospective analysis between depression and hypertension, and therefore describe shortcomings in the operationalization used in the studies from the meta-analysis. By doing this, we describe and therefore reference the 9 included studies individually. Omitting these studies would be a disservice to our argumentation.
2) The availability of data from the Kosovo context is limited, which is why in large part the KOSCO study was established. We therefore reference the most relevant data available from the context, which in many cases date from about 10 years ago.
3) Other older references are from relevant validated instruments and established theories.

The omission of the 9 studies from the meta-analysis would bring the ration down to below 30. We consider the proportion of older references appropriate given the above mentioned points.

**Minor Comments**

2. Line 25: Please revise the sentence "There is growing evidence that depression is an independent risk factor for CVD, yet the underlying mechanisms are not yet elucidated"

It has been revised for clarity.

3. For Table 1: Please follow the proper format for writing the continuous variables mean and SD (e.g., Age (mean ± SD) = 59.4 ± 8.9). Writing frequency (%) and p-values in every row are unnecessary. It can be written in the heading column. For instances

Thank you for this hint. We have updated the table with the suggested format.

4. What is Fisher's exact test p-value and why are not mentioned in this table? Isn't using Fisher's exact test p-value in table 1? There are too many rows with less than 5 frequencies; we should use Fisher's exact test value whether the frequency is less than 5.

We overlooked that municipality was the only variable with more than 20% of cells with a frequency less than 5, so we attempted to conduct a Fishers exact test, but there were too many rows to get an output. We have therefore removed the p value and added a note to the table.

5. Please use a single space in each table.

Single space has now been used in the tables.

6. Line 114: Please write Chi-square or mathematical notations instead of Chi2.

Thank you for this hint. We have changed it to chi-square.

7. Line 122-123: Please insert the reference correctly.

It is not clear to which reference is referred to here. However the manuscript has been read through once again thoroughly and the references corrected where necessary.

8. Line 210: Revise the line. It seems to be repeatedly used Lovibond words.

The reference is indeed by two people named Lovibond.

9. Line 217: Please correct the table number consistency. Table 1 is used repeatedly for a different table and may be table number 2.

The table numbering has been revised carefully and corrected accordingly. Please note a new table 1 was added and the order of the additional tables were changed in the version from December 2022.

10. Please express the confidence interval as (-5.48 – 0.87) (ranging from the lower limit to upper limits) instead of (-5.48, 0.87) and revise all CI values

To avoid confision, we opted to use “to” instead of “-“ given that some of our most important findings have a confidence interval with two negative values. All confidence intervals have been corrected with this format.
